# Coupled Oxides/LLDPE Composites for Textile Effluent Treatment: Effect of Neem and PVA Stabilization

**DOI:** 10.3390/polym12020394

**Published:** 2020-02-09

**Authors:** Norfatehah Basiron, Srimala Sreekantan, Lim Jit Kang, Hazizan Md Akil, Rabiatul Basria S.M.N. Mydin

**Affiliations:** 1 School of Materials & Mineral Resources Engineering, Engineering Campus, Universiti Sains Malaysia, Nibong Tebal, Pulau Pinang 14300, Malaysia; fatehahbasiron17_004@student.usm.my (N.B.); hazizan@usm.my (H.M.A.); 2 School of Chemical Engineering, Engineering Campus, Nibong Tebal, Pulau Pinang Malaysia 14300, Malaysia; chjitkangl@usm.my; 3 Malaysian Institute of Pharmaceuticals and Nutraceuticals (IPHARM), National Institute of Biotechnology Malaysia, Ministry of Science, Technology and Innovation, Bukit Gambir 84400, Malaysia; rabiatulbasria@usm.my

**Keywords:** photocatalyst, stabilization, polymer composites, surface treatment

## Abstract

The polyvinyl alcohol (PVA) and neem extract were grafted onto coupled oxides (3ZT-CO) via reflux process to stabilize the particles to form 3ZT-CO/PVA and 3ZT-CO/Neem. These were then incorporated into LLDPE by melt blending process to give LLDPE/3ZT-CO/PVA and LLDPE/3ZT-CO/Neem composites. The Neem and PVA stabilized particles showed high zeta potential and dispersed homogeneously in water. The stabilization process altered the shape of the particles due to plane growth along the (002) polar direction. The stabilizers acted as capping agents and initiated the one-dimensional growth. The alkyl chain groups from PVA increased the polarity of the LLDPE/3ZT-CO/PVA and played a dominant role in the water adsorption process to activate the photocatalytic activity. This was further enhanced by the homogeneous distribution of the particles and low degree of crystallinity (20.87%) of the LLDPE composites. LLDPE/3ZT-CO/PVA exhibited the highest photodegradation (93.95%), which was better than the non-stabilized particles. Therefore, the photocatalytic activity of a polymer composite can be enhanced by grafting PVA and neem onto couple oxides. The LLDPE/3ZT-CO/PVA composite was further used to treat textile effluent. The results showed the composite was able to remove dye color by 93.95% and to reduce biochemical oxygen demand (BOD) and chemical oxygen demand (COD) by 99.99%.

## 1. Introduction

The textile industry provides significant economic benefits. However, it must also address the environmental and social degradation resulting from its activities. This is associated with a considerable volume of water-soluble waste substance contained in the discharge as a result of the dyeing processes. This discharge contains dye (azoic, disperse, anthraquinone, etc.) and other additives such as surfactants, salts, polymers, and solubilizing agents [1,2]. Normally, these chemicals are rich in color, BOD, and COD level. These discarded chemicals end up in water bodies where they become a serious threat to the ecosystem. 

Lately, treatment of textile waste water using titanium dioxide (TiO_2_) nanoparticles has captured researcher’s attention due to its high thermal stability, biocidal effects, possibility of photodegradation of polymers, and their environmental friendly catalyst properties [3,4]. However, the widespread use of the nanoparticles in real application is hindered due to safety reasons and the difficulty in separating the nanoparticles after the treatment process. Therefore, efforts have already been devoted to immobilize the TiO_2_ nanoparticles on solid media. In addition, the immobilized photocatalyst can be regenerated and used repeatedly. The key problems in the immobilization of nanoparticles in solid media are its tendency to agglomerate and poor dispersion of the particles in the solid matrix due to its polarity difference with the photocatalyst. 

The hydrophilic surface of the metal oxide nanoparticles with high energy tend to aggregate and lead to agglomeration. This causes interfacial incompatibility with the nonpolar polymer matrix [5]. Therefore, the interface between the solid matrix and the nanoparticle is a critical factor that affects the composites performance. It changes the macromolecular state of the polymer (e.g., composition gradient, crystallinity, changed mobility, etc.), thus altering the overall material behavior [6]. The surface modification of metal oxides with polymer has become one of the most used techniques to address agglomeration problem [7,8,9,10]. Polyvinyl alcohol (PVA) is a one of the synthetic petroleum-based polymers. PVA has a carbon–carbon single bond and hydroxyl groups (OH) that are attached to the methane carbon. The OH group forms hydrogen bonds between the polymer and metal oxides [8]. Several recent studies showed that PVA can enhance the distribution of metal oxides in polymer [5], but none focused on dye degradation and effluent treatment to improve BOD, COD level, and to degrade color.

In this study, PVA-stabilized coupled oxide photocatalyst (3ZT-CO), which is more efficiently is proposed to be incorporated into the solid matrix of LLDPE. ZnO was selected to form the heterojunction with TiO_2_ in 3ZT-CO because it is a non-toxic, low cost, and has a wide band gap energy with outstanding optical and electrical properties. In addition, ZnO has potential to kill a wide range of microorganism through the release of Zn^2+^ ions that are responsible for its antimicrobial effects [11]. In an earlier report, it was shown that the formation of heterojunction between TiO_2_ and ZnO can reduce the recombination of electron hole pairs. This can expedite the photodegradation process of dye [12]. 

*Azadirachta indica* also known as neem, is a tropical ever green native tree to India and other Southeast Asian countries [13]. In India, neem has been used in ayurvedic medicine for more than 4000 years and has been called as “the village pharmacy” due to its medicinal properties [14]. Recently, neem extracts were used in the green-synthesis of various nanoparticles like silver, ZnO, gold, etc. [14,15,16,17,18]. This is due to the phytochemicals that are present in the extract. Some of the phytochemicals identified in neem extract are terpenoids and flavanones. These phytochemicals act as reducing agents as well as capping agents and help in stabilizing the nanoparticles [13]. Therefore, the surface of coupled oxides was stabilized with neem extracts through chemical method and the performance was compared with PVA stabilized photocatalyst. The OH groups and the phytochemicals play critical roles in the surface stabilization. Compared to non-stabilized particles, the distribution of the heterojunction particles was much improved after stabilization with PVA or neem. Methylene blue (MB) photodegradation and industrial textile effluent treatment were used to study the performance of PVA and neem stabilized coupled oxide LLDPE composites. This work illustrates a new method to prepare stabilized coupled oxides LLDPE composites with homogenous particle distribution and to degrade the color of MB, BOD, and COD concentration in textile effluent. 

## 2. Materials and Methods 

### 2.1. Materials

Titanium(IV) isopropoxide (TTIP) and zinc acetate dihydrate (ZAD) were purchased from Sigma-Aldrich (Selangor, Malaysia). Ethanol, dichlorobenzene and methylene blue were purchased from Merck Millipore (Selangor, Malaysia). Other chemicals utilized were LLDPE pellet supplied by Exxon Mobil, PVA (Mw: 72,000, Merck, 97.5%, melt index 1.0 g 10 min^−1^, density of 0.918 gcm^−3^) and neem powder. The neem powder was purchased from a local store. 

### 2.2. Synthesis of 3ZT-CO Particles 

The procedure of synthesizing the ZnO/TiO_2_ particles with a 3:1 ratio and designated as 3ZT-CO is given in a previous report [19]. It was prepared via sol–gel method using TTIP and ZAD with a ratio of 3:1. The precipitate was obtained by hydrolysis and condensation process. The precipitate was washed, centrifuged, dried overnight at 80 °C, and calcined for 2 h at 500 °C. 

### 2.3. Stabilization of 3ZT-CO Particles

The 3ZT-CO particles were stabilized according to previously reported study on ZnO [20]. First, the 3ZT-CO was dispersed in 30 mL of deionized water under vigorous stirring for 30 min at room temperature. Simultaneously, PVA was dissolved in 30 mL of deionized water under vigorous stirring for 30 min at 90 °C. Next, the PVA solution was added dropwise to the 3ZT-CO suspension and stirred for 4 h. After the addition, the dispersed mixture was centrifuged, decanted, and oven-dried for 24 h at 50 °C. The sample was labeled as 3ZT-CO/PVA. For neem stabilization, 3ZT-CO was dispersed in 30 mL of ethanol and stirred for 15 min. Approximately 10 wt% of neem extract [21] was added to the mixture. Then, the suspension was refluxed at 55 °C for 6 h in ethanol. The dispersed mixture was centrifuged, decanted, and dried at 60 °C overnight. This sample was labeled as 3ZT-CO/Neem. 

### 2.4. Characterization of 3ZT-CO

The chemical structure and stabilization mechanism of 3ZT-CO were confirmed by Fourier Transform Infrared (FTIR) spectrophotometer (Tensor27, Bruker Optics, Ettlingen, Germany). The shape of 3ZT-CO was analyzed using field emission scanning electron microscope (FESEM-EDX, Zeiss, Supra 35VP, Carl Zeiss NTS GmbH, Oberkochen, Germany). The crystal structure of the stabilized particles was determined using an X-ray diffractometer (XRD). Dynamic light scattering (DLS) and zeta potential were measured with Malvern Zetasizer NanoZS particle analyzer (Malvern Instruments Ltd., Malvern, UK) at a wavelength of 633 nm with a solid-state He–Ne laser at a scattering angle of 173° at 25 °C. Each sample was diluted to 0.1 mg mL^−1^, followed with filtration through a Whatman Anotop nylon membrane with a pore diameter of 220 nm. Compatibility test was tested using 1,4-dichlorobenzene. Stabilized and non-stabilized 3ZT-CO particles were added to the prepared solvent at a ratio of 1:10. The samples were stirred vigorously and left to rest for 20 min and the dispersion was observed after 3 months [22].

### 2.5. Preparation LLDPE Composites

LLDPE composites discs with a diameter of 25 mm and thickness of 0.5 mm were prepared via melt blending method using injection molding with 5 wt% of 3ZT-CO. The injection molding process parameters used are as follows. Temperature profile screw temperature: 165 °C, 175 °C, and 170 °C; injection pressure: 0.2 MPa; cooling time: 3 s; screw rotation: 40 rpm; screw stroke: 8.5 mm. The resulting non-stabilized couple oxides composite was labeled as LLDPE/3ZT-CO. The LLDPE composite of the 5wt% PVA and neem-stabilized samples were labeled as LLDPE/3ZT-CO/PVA and LLDPE/3ZT-CO/Neem, respectively. For comparison, LLDPE without incorporating any oxide was also used.

### 2.6. Characterization of LLDPE Composites

The chemical properties of the LLDPE and LLDPE composites were evaluated using Fourier Transform infrared spectroscopy. Melting properties and degree of crystallinity of the composites were evaluated with Perkin Elmer differential scanning calorimeter (DSC). Each LLDPE composite sample with weight of 10–15 mg was scanned from 30 °C to 150 °C with a heating rate of 10 °C min^−1^ in nitrogen (N_2_) atmosphere (flow rate of 50 mL min^−1^). Then, the melting temperature (Tm) and the enthalpy of fusion (∆H_f_) for the composites were obtained. Microanalysis and elemental mapping analysis were conducted using FESEM images with an EDX detector. 

### 2.7. Photocatalytic Activity

The photocatalytic activity of LLDPE composites (with a disc thickness of 5 mm and a diameter of 25 mm) were evaluated via photodegradation of MB under visible light. The composites were dipped in 40 mL of 10 ppm MB solution. The set-up was placed in the dark condition for 48 h to attain an equilibrium adsorption state. Subsequently, the MB solution was irradiated with visible light for 120 h. A 4.0 mL dye solution was withdrawn every 12 h interval and measured using UV–vis spectroscopy at 664 nm. The percentage of dye degradation was calculated using Equation (1).

(1) $$Degradation\left(\%\right)=\frac{C_{o}-C_{t}}{C_{o}}X100$$

where *C*_o_ is the initial MB concentration and *C*_t_ is the MB concentration at time t. 

### 2.8. Color Degradation, Biochemical Oxygen Demand (BOD), and Chemical Oxygen Demand (COD) Analysis

The LLDPE/3ZT-CO/PVA composite with high MB degradation was evaluated to treat textile effluent collected from Ayu Fashion Sdn Bhd, a textile factory in Kelantan, Malaysia. Effluent that was collected after the filtration system suffer from high BOD, COD levels, and dye color. The effluent was collected and treated with LLDPE/3ZT-CO/PVA composite for 6 h and then the sample was sent to MyCO_2_ laboratory to quantify the BOD, COD and color concentration. Experiments were carried out without pH control under non-sterilized condition. The effluent were analyzed in MyCO_2_ laboratory based on the American Public Health Organization (APHA) method [23]. 

## 3. Results and Discussions

### 3.1. Crystal Structure. Morphology, and Chemical Mechanism of Functionalized Particles

**Figure 1:**
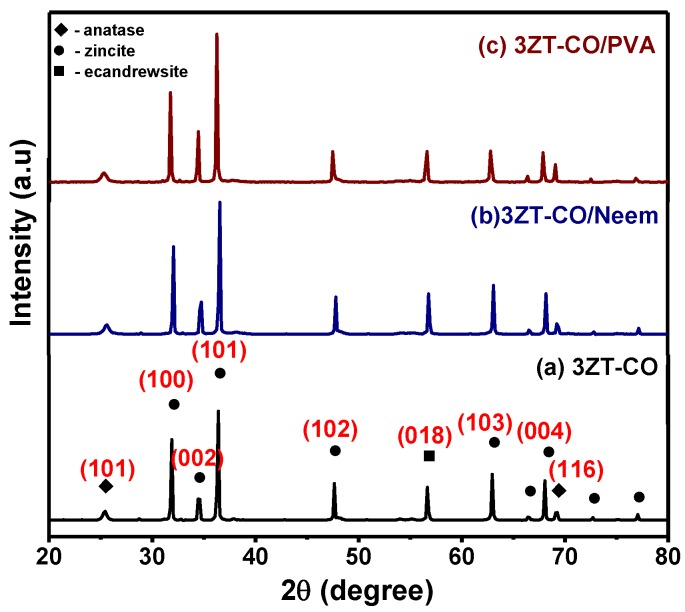
The XRD pattern of (**a**) the non-functionalized nanoparticles, 3ZT-CO, and the stabilized nanoparticles (**b**) 3ZT-CO/Neem and (**c**) 3ZT-CO/PVA.

The XRD patterns of stabilized and non-stabilized 3ZT-CO particles are presented in Figure 1. The result of the non-stabilized 3ZT-CO particles (Figure 1a) showed three different phases: zincite, anatase, and ecandrewsite. The peaks at 2θ = 25.36° and 69.18° were assigned to (101) and (116) of anatase (JCPDS 731764), respectively. Whereas, the peaks at 2θ = 31.79°, 34.53°, 36.36°, 47.64°, 62.96°, 66.53°, 68.05°, 72.66°, and 77.05° are attributed to (100), (002), (101), (102), (103), (200), (112), (004), and (202) of zincite (JCPDS 361451), respectively. A peak at 56.69° corresponds to the (018) plane of ecandrewsite (JCPDS 261500). The ecandrewsite (ZnTiO_3_) phase can only be observed when a proper stoichiometry ratio reaction between ZnO and TiO_2_ was carried out to form ZnTiO_3_. As calcination was done only at 500 °C in this study, the formation of (ZnTiO_3_) can be considered to be premature or incomplete [24]. This explains the low intensity of (ZnTiO_3_) peaks compared to the anatase and zincite peaks. The diffractograms of the 3ZT-CO/Neem and 3ZT-CO/PVA shown in Figure 1b,c reflects similar pattern as in non-stabilized particles. This suggests that stabilization by neem or PVA has insignificant effect in the phase transformation [20]. However, the intensity of (002) plane for 3ZT-CO/Neem (Figure 1b) and 3ZT-CO/PVA (Figure 1c) gradually increased compared to other phases. This signifies particle growth along the [002] polar direction. This observation reveals neem and PVA could be used as a capping agent to induce c-direction growth. The crystallite size can be estimated using the Scherrer Equation [25],

D = Kλ/βcos(θ)(2)

where D is the average crystallite size, K is the Scherrer constant, λ is the wavelength of the X-ray (1.5408 nm) used, θ is the Bragg diffraction angle, and β is the full width at half maximum (FWHM). Therefore, the calculated average crystallite size for 3ZT-CO is 60.67 nm, 3ZT-CO/Neem is 60.65 nm and 3ZT-CO/PVA is 60.70 nm. It was found that the stabilization process by neem and PVA did not affect the crystallite size. 

**Figure 2:**
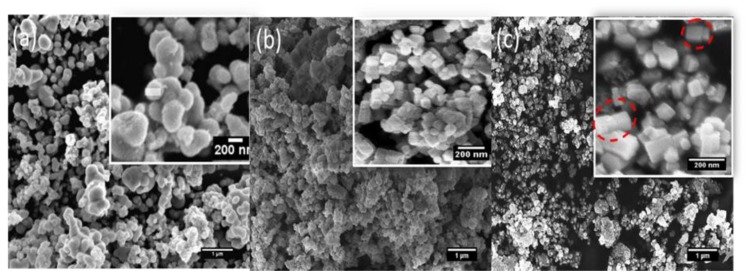
The SEM images of (**a**) 3ZT-CO, (**b**) 3ZT-CO/Neem, and (**c**) 3ZT-CO/PVA.

**Figure 3:**
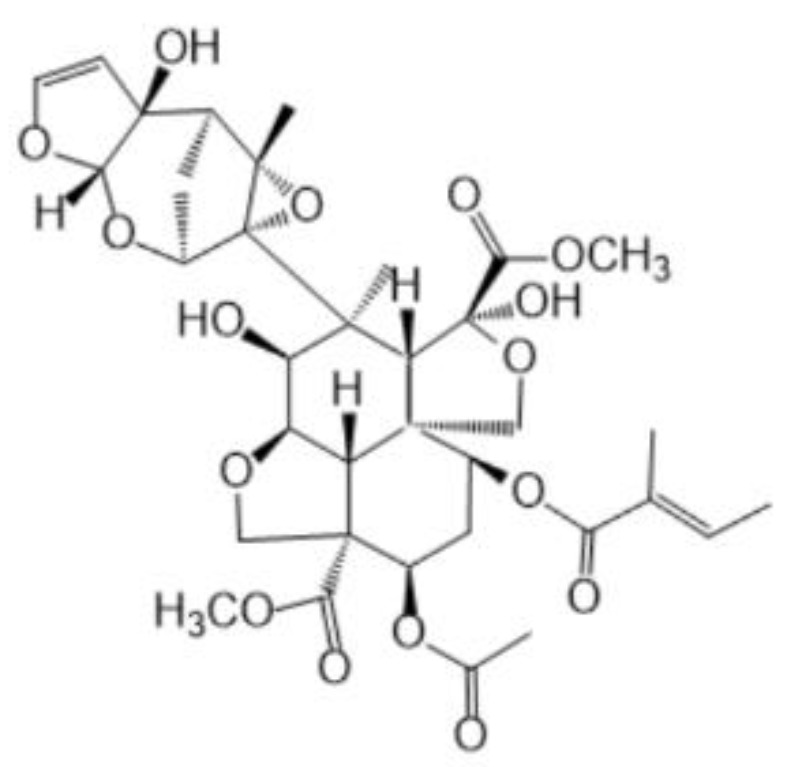
The structure of neem compound.

The morphology of 3ZT-CO, 3ZT-CO/Neem, and 3ZT-CO/PVA were examined by SEM and the results are illustrated in Figure 2. The SEM micrograph of 3ZT-CO (Figure 2a) consists of quasi-spherical-like particles that are heavily agglomerated. After grafting with the neem extract, the morphologies were marginally elongated to form square faced particles. (Figure 2b). Theoretically, azadirachtin is a major component of neem [16] that contain active ingredients of limonoids. The generalized structure of this component is shown in Figure 3, which indicate the presence of abundant hydroxyl (OH) and carbonyl (C=O) groups. When 3ZT-CO was mixed with neem extracts in ethanol, the leached Zn^2+^ ions will distribute uniformly and form a three dimensional network structure with limonoids which has OH, C=O, C–O, and epoxy groups [13]. The resulting network will undergo further reaction with 3ZT-CO, thus growing into elongated square shaped particles. For 3ZT-CO/PVA the morphologies have prominent facet structure forming hexagonal shaped rods as presented in Figure 2c. In certain regions, interfaces could be found between ZnO nanorods, suggesting stacking at (0 0 2) polar surface. This is in agreement with the work reported by Pung et. al, who suggested stacking at (0 0 2) the polar surface originate from positively charged Zn^2+^—terminal surface or negatively charged O^2−^—terminal surface [26]. These two oppositely charged polar surfaces are inclined to join in order to reduce the total surface energy. The diameter of ZnO nanorods were found to be in the range of 30 nm to 60 nm and had a length of 100 to 300 nm. The agglomeration of particles was further reduced after stabilization with PVA and neem. This was due to the effect of steric hindrance of the PVA and neem structure 8.

**Figure 4:**
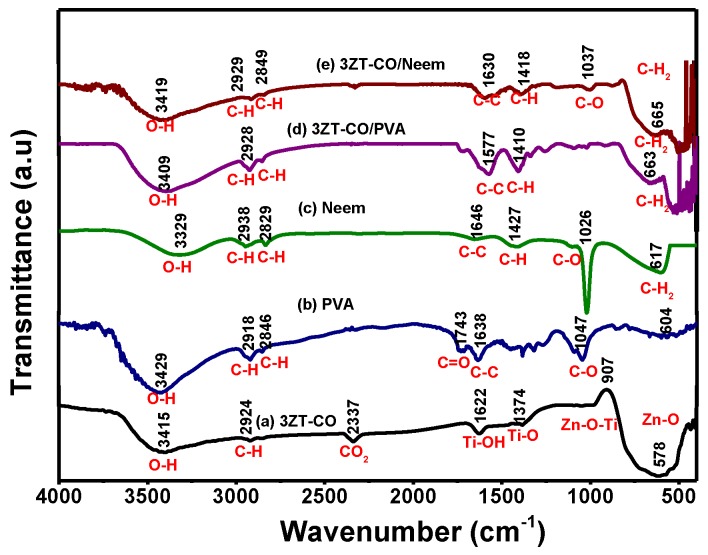
The FTIR spectra of the prepared samples (**a**) 3ZT-CO, (**b**) PVA, (**c**) neem extract, (**d**) 3ZT-CO/PVA, and (**e**) 3ZT-CO/Neem.

**Figure 5:**
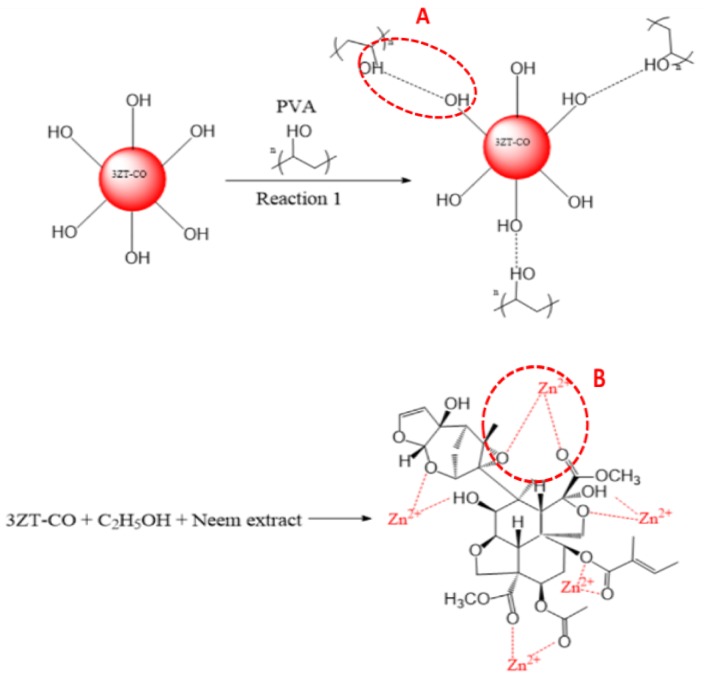
(**A**) PVA grafting on 3ZT-CO particle. (**B**) Stearic stabilization by neem in 3ZT-CO/Neem.

The surface functional group of 3ZT-CO, 3ZT-CO/PVA, and 3ZT-CO/Neem were compared with the pure stabilizing agents and the results are shown in Figure 4. For 3ZT-CO particles (Figure 4a), the broad peak of Zn-O stretching vibration is observed at 578 cm^−1^ [26]. However, the transmission bands associated with Ti-O at 1374 cm^−1^ and Ti-OH at 1622 cm^−1^ are not apparent, indicating that TiO_2_ might have reacted with ZnO to form Zn-O-Ti. This is affirmed by the appearances of a broad intense band at 500 to 800 cm^−1^. This result is in agreement with the absence of titania phase and the presence of zincite and ecandrewsite phases in the XRD analysis (Figure 1a). The band at 2924 cm^−1^ is associated to the C–H stretching vibration that originated from organic impurities of zinc hydroxy acetate complex or tetra nuclear oxo zinc acetate cluster (Zn_4_O (CH_3_COO)_6_). The broad and less intense bands observed at 3415 cm^−1^ and 2337 cm^−1^ are assigned to water (OH) on the 3ZT-CO surface, originating from solvent and CO_2_ stretching mode, respectively [24]. Figure 4b shows pure PVA consists of a broad intense band at around 3429 cm^−1^, corresponding to the prominent –OH stretching. Other than that, the asymmetric and symmetry C-H (CH_2_) stretching, C-C stretching, and CH_2_ bending vibration bands were observed at 2918 cm^−1^, 2846 cm^−1^ [27], 1638 cm^−1^, and 604 cm^−1^, respectively. These four bands are ascribed to the alkyl chains of PVA. The band at 1047 cm^−1^ is due to the—CO stretching vibration. The band at 1743 cm^−1^ was due to the presence of the excess acetate group from the synthesis of PVA by the hydrolysis of polyvinyl acetate [27]. For neem (Figure 4c), a shallow broad band appeared around 3329 cm^−1^ attributed to less –OH stretching compared to PVA. Besides, other bands were observed at 2938 cm^−1^, 2829 cm^−1^, 1646 cm^−1^, 1427 cm^−1^, 1026 cm^−1^, and 617 cm^−1^, which are attributed to the symmetric and asymmetric CH_2_ stretching, C-C bending, C-H bending, C-O, and CH_2_ bending originating from neem and ethanol. For the 3ZT-CO/PVA (Figure 4d), the –OH stretching was shifted from 3415 cm^−1^ to 3409 cm^−1^ due to the adsorption of PVA on the particle surface via hydrogen bond as demonstrated schematically in Figure 5A. An oxygen in the OH group of the coupled oxides form hydrogen bond with the OH group of PVA (the area labeled as “A” in reaction (1)). Thus, the vibration of –OH stretching became low. Other bands appearing at 2928 cm^−1^, 1577 cm^−1^, 1410 cm^−1^, 663 cm^−1^, and 520 cm^−1^ corresponds to the presence of C-H, C-C, C-H, and Zn-O bands which further affirm the bonding within the alkyl chain of PVA and ZnO particles, thus inducing stabilization. As for 3ZT-CO/Neem (Figure 4e), the intensity of the OH stretching band at 3419 cm^−1^ had increased compared to the pure particles attributed to the synergy effect of free OH groups from neem and the particles. Besides, the sharp band at 1026 cm^−1^ in neem disappeared [27]. This suggests the formation of bridges between OH and ligand groups from neem with Zn^2+^, labeled as B in reaction 3 (Figure 5A). Additionally, more bands were observed at 2929 cm^−1^, 2849 cm^−1^, 1630 cm^−1^, 1418 cm^−1^, 1037 cm^−1^, and 665 cm^−1^ which was attributed to the presence of symmetric and asymmetric stretching of C-H, C-C, C-H, C-O-, and CH_2_ groups.

### 3.2. Stability and Compatibility Test of the 3ZT-CO Particles

**Figure 6:**
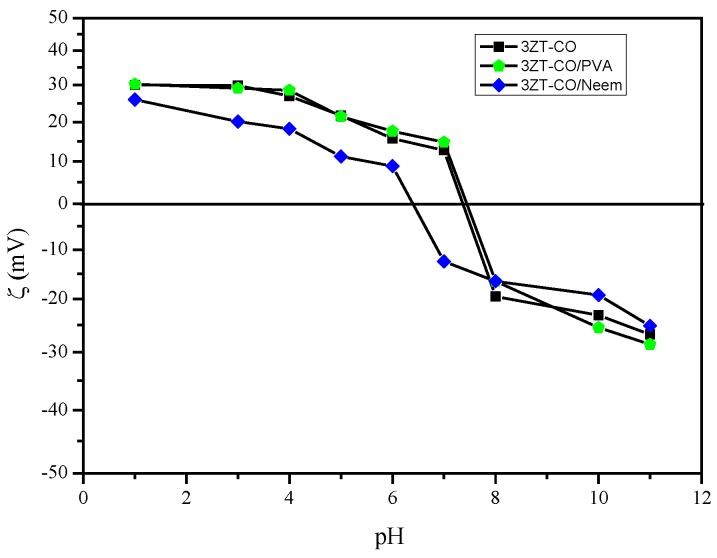
The graph of zeta potential versus pH of non-functionalized ■3ZT-CO, stabilized ⬟3ZT-CO/PVA, and ◆3ZT-CO/Neem at different pH values.

The zeta potential was measured at each pH to determine the surface charge. The 3ZT-CO has a zeta potential of +30.1 to −26.75 mV between pH 1 to 11. Figure 6 shows the isoelectric point of 3ZT-CO at pH 7.35. This affirms the presences of OH groups on the 3ZT-CO surface, which exhibit the amphoteric behavior [8]. Therefore, the surface charge can be easily changed under acidic or alkaline conditions in aqueous solution. The OH groups are open for deprotonation, capable to form hydrogen bonding and have electrostatic effect with suitable compounds [28]. Therefore, incomplete O-coordinated Zn, Ti ions (Lewis acids) can occur on coupled oxides. Based on this theory, it can be assumed that coupled oxides have the potential to form hydrogen bonding with PVA macromolecules coated on 3ZT-CO. The zeta potential of 3ZT-CO/PVA was found to be +30.23 to −28.56 mV between pH 1 to 11, and its isoelectric point was at pH 7.44. The difference in the isoelectric point between these two particles could be due to three factors: (i) the positional shift in the shear plane, (ii) surface charge alterations caused by PVA adsorption, and (iii) the obstruction of the active sites of Zn-Ti-O due to adsorbed polymer macromolecules [29]. The zeta potential of 3ZT-CO/Neem varied considerably with pH. This result implies that OH and C=O groups are loosely bound to the surface of 3ZT-CO/Neem particles. The C=O group with negative surface charge results in high negative zeta potential that contribute to improved dispersion of the particles. This is represented in the particle size distribution, which was measured by DLS. The particle size distribution was calculated by using Stokes–Einstein equation [30]:

*d*(*H*) = *kT*/3*πηD* (3)

where *d*(*H*) is the hydrodynamic diameter, *D* is the translational diffusion coefficient, *k* is Boltzmann’s constant, *T* is absolute temperature, and *η* is the viscosity. The average hydrodynamic size of 3ZT-CO was 120.12 nm, 3ZT-CO/PVA was 110.34 nm and 3ZT-CO/Neem was 96.21 nm. This result suggests that surface stabilization with PVA and Neem had reduced agglomeration of coupled oxides. This improvement in particle dispersion occurred due to the long and branched alkyl chains that induced substantial steric hindrance to repel the particles [31,32]. Furthermore, Smith et al. reported that zeta potential does not have a significant effect on repulsive electrostatic interaction to stabilize catalysts in nonpolar solvent. This was because nonpolar solvent has low dielectric value and high ionization energy [33]. 

**Figure 7:**
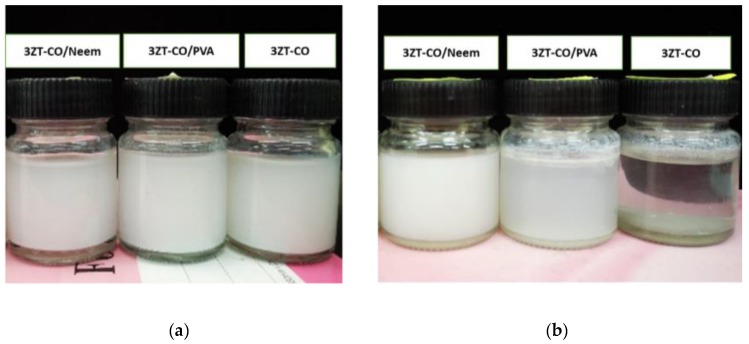
Compatibility test: (**a**) Various nanoparticles dispersed in 1,4-dichlorobenzene and (**b**) 3ZT-CO aggregates in 1,4-dichlorobenzene after 3 months.

The compatibility test of the samples in 1,4-dichlorobenzene (Figure 7) illustrate the colloidal stability of particles in a nonpolar medium. Figure 7a shows all the samples dispersed in 1,4-dichlorobenzene after sonication. It shows that the samples are well dispersed in the solvent. However, after 3 months, (Figure 7b), sedimentation of 3ZT-CO took place showing that non-stabilized particles (3ZT-CO) which are hydrophilic has no interaction with nonpolar solvent. Thus, forming larger aggregates and settle down at the bottom. This result is in agreement with the measurement of the hydrodynamic diameter as tabulated in Table 1. 3ZT-CO/PVA and 3ZT-CO/Neem (Figure 7b) did not agglomerate after 3 months in 1,4-dichlorobenzene. This confirms that the 3ZT-CO particles are well stabilized and stay dispersed in nonpolar media; demonstrating an excellent colloidal stability. As reported by Hofmann et al., the extension of the ligands is an important factor for nanoparticle stearic stabilization. A bulky ligand often leads to higher stability of the nanoparticle–ligand system [34]. 

### 3.3. Investigation on Chemical, Crystallinity, and Particle Distribution of Polymer Composites

**Figure 8:**
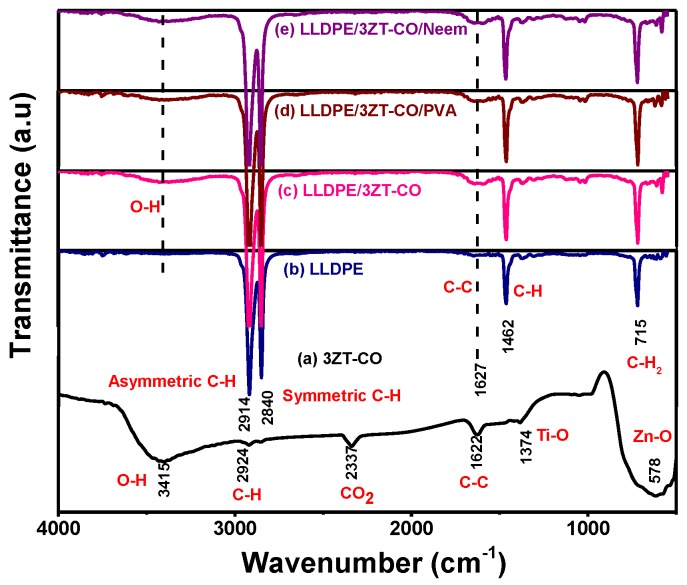
The FTIR spectra of (**a**) 3ZT-CO, (**b**) LLDPE (**c**) LLDPE/3ZT-CO, (**d**) LLDPE/3ZT-CO/PVA, and (**e**) LLDPE/3ZT-CO/Neem.

The FTIR, DSC, and elemental mapping were used to study the chemical, crystallinity and morphology of the coupled oxide and the LLDPE composites. The FTIR spectra of 3ZT-CO particles, the LLDPE and LLDPE composites are illustrated in Figure 8. The FTIR spectra for LLDPE and LLDPE composites (Figure 8a–e) showed transmission bands at 2914 cm^−1^, 2840 cm^−1^, 1627 cm^−1^, 1462 cm^−1^, and 715 cm^−1^, corresponding to asymmetric and symmetric C-H stretching, C-C stretching, C-H, and CH_2_ bending of the LLDPE structure [35]. However, for LLDPE composites showed the OH band as shown in Figure 8b–e, confirming the successful incorporation of coupled oxide complexes into the LLDPE matrix. The presence of OH band shows LLDPE composites have higher polarity than the LLDPE [36], which act as a driving force for water uptake and will expedite the release of reactive oxygen species (ROS) for photodegradation [37]. 

DSC analysis was used to study the crystallization behavior of LLDPE and LLDPE composites. The results are shown in Table 2. The degree of crystallinity of LLDPE and LLDPE composites are calculated by assuming a theoretical value of 288 J/g [38] for the heat involved in the fusion of a completely crystalline LLDPE polymer by using the following Equation [39]:

(4) $$\mathrm{Xc}\;=\;\frac{\Delta Hf}{\Delta H_{100}\left(1-x\right)}\;\times \;100$$

Melting point and ∆H_m_ of LLDPE composites are lower than LLDPE. Furthermore, the degree of crystallinity dropped from 31.89% to 20.87% with stabilized 3ZT-CO. This is because of the interruption of spherulite growth by immiscible coupled oxides and steric hindrance caused by the stabilization process to form crystalline structure [40]. The high amount of amorphous structure was expected to enhance the water uptake of LLDPE composites thus, expedite metal ion release and reactive oxygen species (ROS) production [41] to improve photodegradation activity. 

**Figure 9:**
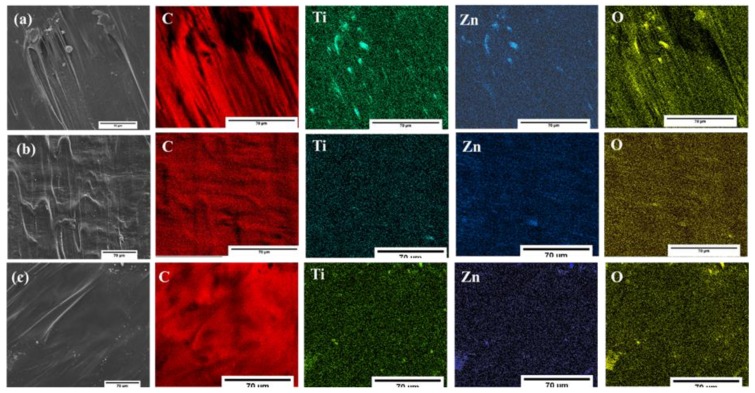
The elemental mapping of LLDPE composites (**a**) LLDPE/3ZT-CO, (**b**) LLDPE/3ZT-CO/PVA, and (**c**) LLDPE/3ZT-CO/Neem.

Figure 9 shows the morphology images and distribution of the elements (C, Ti, Zn, and O) within the polymer matrix. The colored elemental mapping images show that C (red), Ti (green), Zn (blue), and O (yellow) atoms were homogeneously distributed over the entire LLDPE matrices. Table 3 reveals the atomic ratio for all LLDPE composites. All the composites showed a similar trend of ~3:1 ratio. However, clusters with micrometer diameters were visible at various spots (Figure 9a), for non-stabilized particles attributed to the agglomeration of hydrophilic particles in the polymer matrix. In contrast, the agglomeration and distribution of coupled oxides was improved after stabilization, as showed in Figure 9b,c.

### 3.4. Visible Light Photocatalytic Reactivity, Wastewater Treatment, and Mechanism

**Figure 10:**
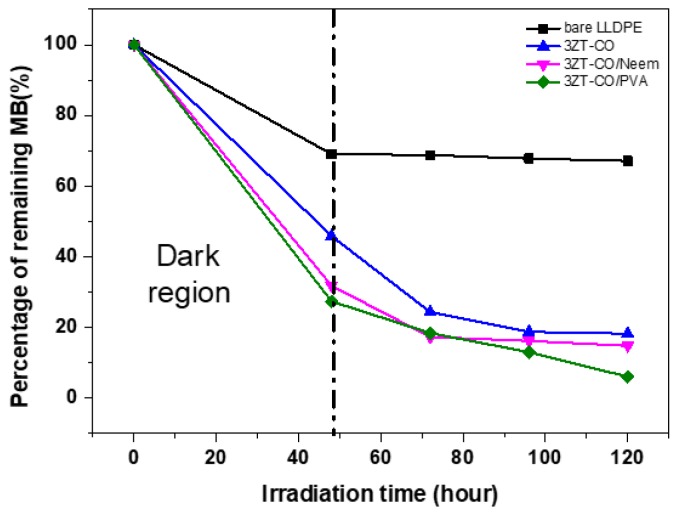
The photocatalytic activity of LLDPE and its composite films on the discoloration of MB under visible light irradiation as a function of degradation time after 48 h.

Figure 10 presents the photocatalytic activity of LLDPE and LLDPE composites under visible light radiation. Evidently, the adsorption of MB after 48 h differed significantly for LLDPE and LLDPE composites. LLDPE/3ZT-CO/PVA showed the highest adsorption percentage (65%), followed by LLDPE/3ZT-CO (50%) and LLDPE/3ZT-CO/Neem (61%). This clearly indicates that the hydrophilic nature of the coupled oxides and the high amorphous region in LLDPE composites allowed better dye molecule adsorption on the LLDPE matrix. Upon irradiation with visible light, the dye degradation increased to 93.95%, 89.25%, and 79.00% for the LLDPE/3ZT-CO/PVA, LLDPE/3ZT-CO/Neem and LLDPE/3ZT-CO, respectively. The high amorphous region, homogenous distribution of coupled oxides and the presence of OH groups in the LLDPE/3ZT-CO/PVA are the important factors that influence the photocatalytic (PC) efficiency. These factors enhance the rate of water and dissolved oxygen diffusion [42] via LLDPE matrix to accelerate the release reactive oxygen species (ROS) for PC activity. Although, LLDPE/3ZT-CO/Neem showed similar crystallinity with LLDPE/3ZT-CO, the PC performance was much better than non-stabilized coupled oxides. This is due to the high OH content of neem ligands that enhance the water uptake for PC activity. 

The suggested mechanism for the photodegradation process of MB dye is as follows: Firstly, the molecules of MB dye are adsorbed on the LLDPE/3ZT-CO/PVA. Upon light irradiation with suitable wavelength, the photocatalyst will excite electrons (e^−^) from the valence band to the conduction band, creating positive holes (h^+^) in the valence band. Then, these two radicals (e^−^ and h^+^) will react with the adsorbed water molecules (H_2_O) and dissolved oxygen (O_2_), producing free hydroxyl radicals (OH) and superoxide anion radicals (O_2_). This is expedited by the functionalized hydrophilic 3ZT-CO. Apart from that, the larger amorphous region in the composite accelerate the ROS release, thus oxidizing the MB molecules and decompose them to benzenes ring compounds. Upon complete oxidation, these compounds will eventually convert to water and carbon dioxide [43]. 

To investigate the potential application of LLDPE composites for water treatment, the LLDPE/3ZT-CO/PVA was used to treat dye effluents discharged from textile industry. Table 4 shows the raw wastewater effluent characteristics as compared to the treated one. The average values of BOD and COD in the untreated effluent were 5506.0 and 19,968.9, respectively. After photo oxidation treatment using LLDPE/3ZT-CO/PVA, the average value of BOD and COD dropped to 11.0 and 49.0 mg/L, respectively. This excellent COD removal efficiency was attained due to the ability of the LLDPE composite to degrade the organic pollutants to smaller molecules [44]. The BOD was reduced by 99.9% after treatment, indicating that the amount of ROS required to oxidize the organic compounds to carbon dioxide and water is high and able to degrade the recalcitrant dye in the effluent [44]. The color of the textile effluent was also improved by 93.40%. In summary, the characteristics after the photo-oxidation treatment using LLDPE/3ZT-CO/PVA showed significant improvement in the effluent discharge quality. 

## 4. Conclusions

The 3ZT-CO with PVA and Neem stabilization helps to reduce the nanoparticle aggregation. This prevents nonhomogenous dispersion of the coupled oxides in the polymer matrix. The stabilization of the catalyst affects the degree of crystallinity and the presence of OH groups, which control the water uptake and ROS release for photo-oxidation activity. LLDPE/3ZT-CO/PVA has the highest MB degradation under visible light irradiation It also satisfy the standard guidelines of effluent discharge quality for BOD and COD by 99% while the color change was 93.40%. These findings suggest a new direction for immobilization of photocatalyst in polymer matrix for textile effluent treatment. 

## Figures and Tables

**Table 1:** The zeta potential and hydrodynamic size of the samples at pH 7.

Sample	Hydrodynamic Size (nm)	Zeta Potential (mV)
3ZT-CO	120.12	+12.8
3ZT-CO/PVA	110.34	+14.7
3ZT-CO/Neem	96.21	−18.5

**Table 2:** The degree of crystallinity profile of LLDPE and LLDPE composites.

Sample	T_m_ (°C)	∆H_m_ (J/g)	∆H_f_° (J/g)	Xc (%)
**LLDPE**	124.26	91.84	288.00	31.89
**LLDPE/3ZT-CO**	122.96	78.75	288.00	27.34
**LLDPE/3ZT-CO/Neem**	122.71	77.70	288.00	26.98
**LLDPE/3ZT-CO/PVA**	122.29	60.10	288.00	20.87

**Table 3:** The elemental mapping analysis of LLDPE composites by EDX.

Sample	Elemental (C) Quantification		Elemental\(Ti) Quantification		Elemental (Zn) Quantification		Elemental (O) Quantification		Relative Atomic Ratio
	Wt (%)	Atomic (%)	Wt (%)	Atomic (%)	Wt (%)	Atomic (%)	Wt (%)	Atomic (%)	Zn:Ti
**LLDPE/3ZT-CO**	94.27	97.38	0.76	0.26	3.53	0.89	1.44	1.48	3.4:1
**LLDPE/3ZT-CO/PVA**	97.23	98.23	0.38	0.17	1.55	0.50	0.84	1.11	2.9:1
**LLDPE/3ZT-CO/Neem**	97.27	98.65	0.24	0.11	1.54	0.36	0.95	0.86	3.2:1

**Table 4:** Comparison of effluent parameters before and after treatment with LLDPE/3ZT-CO/PVA.

Test Description	Unit	Specification	Results		
			Before	After	Efficiency (%)
BOD-5 days test @ 20′C	mg/L	50	5506	11	99.99
Colour (ADMI)	ADMI	200	6940	458	93.40
COD- Chemical Oxygen Demand	mg/L	200	19,968.9	49.0	99.99
